# Draft genome sequence of “*Candidatus* Phytoplasma australasiaticum” strain TBB-AP associated with tomato big bud disease in India

**DOI:** 10.1128/mra.00033-26

**Published:** 2026-03-23

**Authors:** Nian-Pu Li, Shivani Gupta, Sesha Kiran Kollipara, Ting-Hsuan Hung, Govind P. Rao, Chih-Horng Kuo

**Affiliations:** 1 Institute of Plant and Microbial Biology, Academia Sinica 71559, Taipei, Taiwan; 2 Department of Plant Pathology and Microbiology, National Taiwan University 33561, Taipei, Taiwan; 3 Division of Plant Pathology, Indian Agricultural Research Institute, Pusa Campus 28802, New Delhi, India; 4 Horticultural Research Station, Dr YSR Horticultural University 254192, Venkataramannagudem, Andhra Pradesh, India; University of Maryland School of Medicine, Baltimore, Maryland, USA

**Keywords:** genome, phytoplasma, plant pathogens, mollicutes

## Abstract

We report the draft metagenome-assembled genome (MAG) of “*Candidatus* Phytoplasma australasiaticum” strain TBB-AP, obtained from a symptomatic tomato plant collected in Andhra Pradesh, India. This assembly provides a genomic resource for functional and evolutionary studies of phytoplasmas associated with tomato big bud disease.

## ANNOUNCEMENT

Phytoplasmas are wall-less bacteria that inhabit the phloem tissue of infected plants and are transmitted by phloem-feeding insect vectors. These pathogens infect several hundred plant species worldwide, and approximately 50 “*Candidatus* Phytoplasma” species have been formally described to date (1). Solanaceous vegetable crops are susceptible to infection by multiple phytoplasma lineages, among which tomato big bud (TBB) disease represents a significant threat to tomato production globally (2). In India, TBB has emerged as an increasingly important disease (3). To expand genomic resources for TBB-associated phytoplasmas, we generated a metagenome-assembled genome (MAG) of a phytoplasma strain designated TBB-AP, derived from a tomato plant showing big bud symptoms collected on 5 April 2024 in Andhra Pradesh, India (GPS coordinates: 13.834556 N 78.278194 E).

The workflow followed our previously established phytoplasma genome analysis pipelines (4, 5). Unless stated otherwise, methods followed the cited references, kits were used according to the manufacturers’ instructions, and bioinformatics tools were run with default settings. Briefly, total DNA was extracted from midribs of tomato leaves using the DNeasy Plant Mini Kit (Qiagen 69104), followed by library preparation with the KAPA HyperPlus Kit (Roche 07962428001). Sequencing on an Illumina NovaSeq 6000 platform generated 47,444,076 paired-end reads of 150 bp, totaling 14.2 Gb. Illumina reads were quality trimmed using Trimmomatic v0.39 (6). The *de novo* metagenome assembly was performed using Velvet v1.2.10 (7) with a k-mer length of 61 and a minimum contig length of 2,000 bp. Putative phytoplasma contigs were identified by BLASTN v2.11.0 (8) searches against known phytoplasma sequences in GenBank (9) with an e-value cutoff of 1e-15. To assess sequencing depth, Illumina reads were mapped to the contigs using Burrows-Wheeler Alignment (BWA) tool v0.7.17 (10). Gene prediction was conducted using RNAmmer v1.2 (11), tRNAscan-SE v1.3.1 (12), and Prodigal v2.6.3 (13). Protein-coding genes were initially annotated based on homologous genes in “*Candidatus* Phytoplasma luffae” (14) identified using OrthoMCL v1.3 (15), followed by manual curation using BLASTP (8) searches against the GenBank non-redundant database (9) and the KEGG database (16).

The draft MAG of strain TBB-AP consists of 33 contigs with a total length of 558,801 bp, an N50 of 26,969 bp, and an average G+C content of 23.7%. The mapped Illumina reads provided approximately 225.0-fold coverage. Genome annotation identified one complete rRNA operon, 17 tRNA genes, and 471 protein-coding genes. Quality assessment using CheckM2 v1.1.0 (17) estimated 81.8% completeness and 0.1% contamination.

Based on the full-length 16S rRNA gene extracted from the assembly, *i*PhyClassifier (18) classified strain TBB-AP as a member of the 16SrII-D group. Whole-genome comparison using FastANI v1.33 (19) indicated that TBB-AP shares 86.9% alignment fraction and 99.6% average nucleotide identity (ANI) with PR08 (GCA_015239935.3), the reference strain of ‘*Candidatus* Phytoplasma australasiaticum’ (20). Based on the 95% ANI threshold suggested for delineating phytoplasma species, TBB-AP was identified as a member of “*Candidatus* Phytoplasma australasiaticum.” This MAG provides a useful resource for functional and evolutionary studies of TBB-associated phytoplasmas.

## Data Availability

This genome sequencing project has been deposited in the National Center for Biotechnology Information (NCBI) and is publicly available under BioProject PRJNA1336984. The Illumina reads and assembly are available under the accessions SRR35731522 and JBSQTC000000000, respectively.
